# Supplementary material for: Unique transcriptome signatures and GM-CSF expression in lymphocytes from patients with spondyloarthritis
Source: Nat Commun. 2017 Nov 15;8:1510. doi: 10.1038/s41467-017-01771-2 (PMC5688161; doi:10.1038/s41467-017-01771-2)
Supplement: Supplementary file 1 — Supplementary Information [file 41467_2017_1771_MOESM1_ESM.pdf]

Supplementary Figure 1. Gating strategy for ex-vivo flow cytometry phenotype analysis and percentage of cytokine positive cells

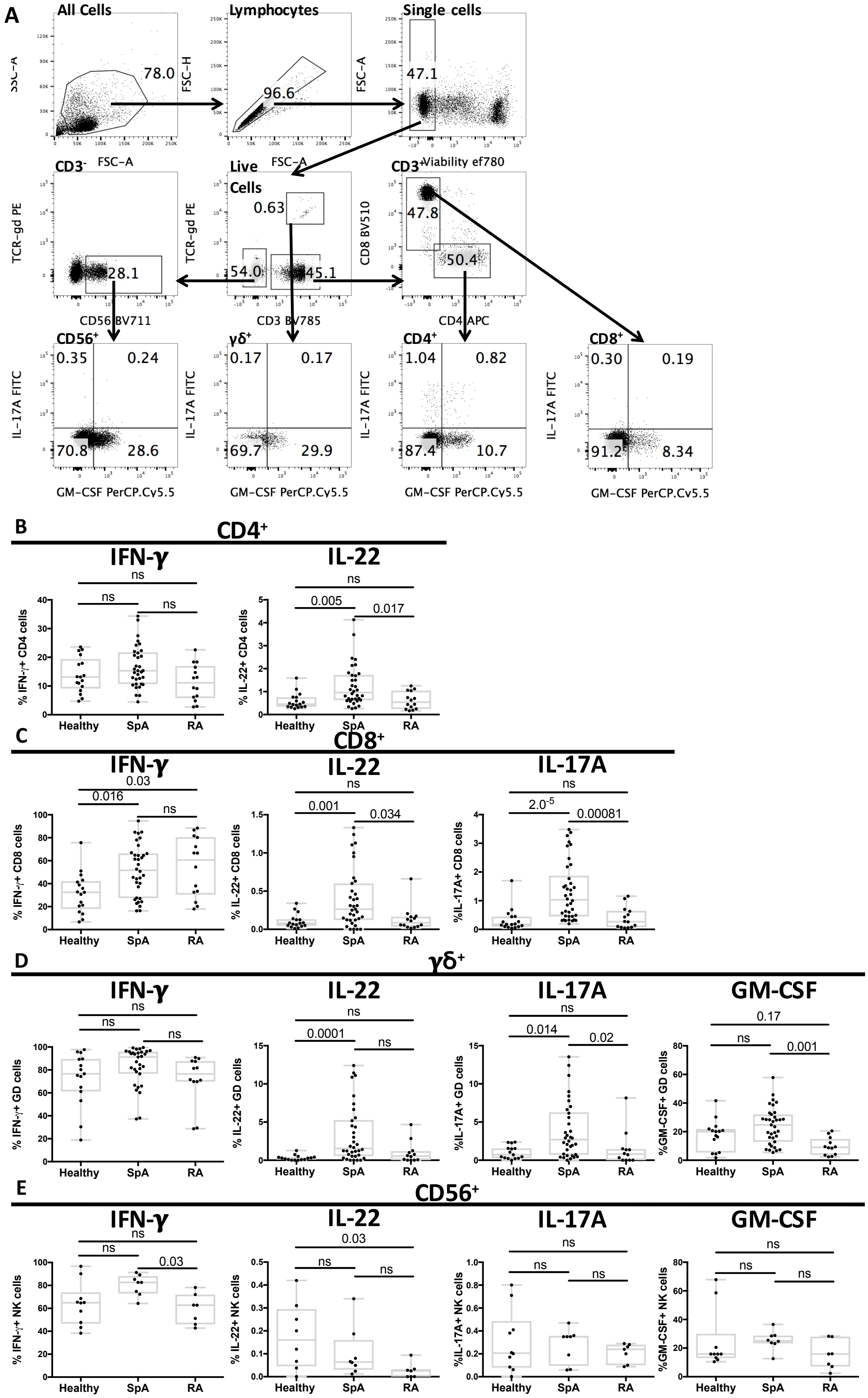

Supplementary Figure 1. Gating strategy for ex-vivo flow cytometry phenotype analysis and percentage of cytokine positive cell. **A** Representative flow cytometry plots from SpA PBMC. All events were gated on Lymphocytes, single cells, then live cells (top row of plots). Live cells were then gated on $CD3^{+}\gamma\delta^{+}$, $CD3^{+}\gamma\delta^{-}$, and $CD3^{-}\gamma\delta^{-}$. $CD3^{+}\gamma\delta^{-}$ cells were then gated on $CD4^{+}$ and $CD8^{+}$. $CD3^{-}\gamma\delta^{-}$ cells were gated on $CD56^{+}$. Therefore the final four subtypes for analysis were $CD3^{+}\gamma\delta^{+}$, $CD3^{+}\gamma\delta^{-}CD4^{+}$, $CD3^{+}\gamma\delta^{-}CD8^{+}$ and $CD3^{-}\gamma\delta^{-}CD56^{+}$. Bottom row of plots shows IL-17A and GM-CSF staining for each of the four subtypes. Unstimulated controls were used to draw the cytokine gates. **B to E** Ex-vivo stimulated PBMCs from patients with SpA RA and healthy controls (HC) were gated on $\gamma\delta^{-}CD3^{+}CD4^{+}$ (**B**, SpA=38, RA=14, HC=17), $\gamma\delta^{-}CD3^{+}CD8^{+}$ (**C**, SpA=34, RA=12, HC=15), $CD3^{+}\gamma\delta^{+}$ (**D**, SpA=34, RA=12, HC= 15) and $CD3^{-}CD56^{+}$ (**E,** SpA=8, RA=7, HC=10) and the percentage of positive for intracellular cytokines was determined. Statistical significance was determined using a Kruskal-Wallis test with p values calculated using Dunn's multiple comparisons test.

Supplementary Figure 2. CD4 GM-CSF frequency in SpA patients by comorbidity and TNFi treatment

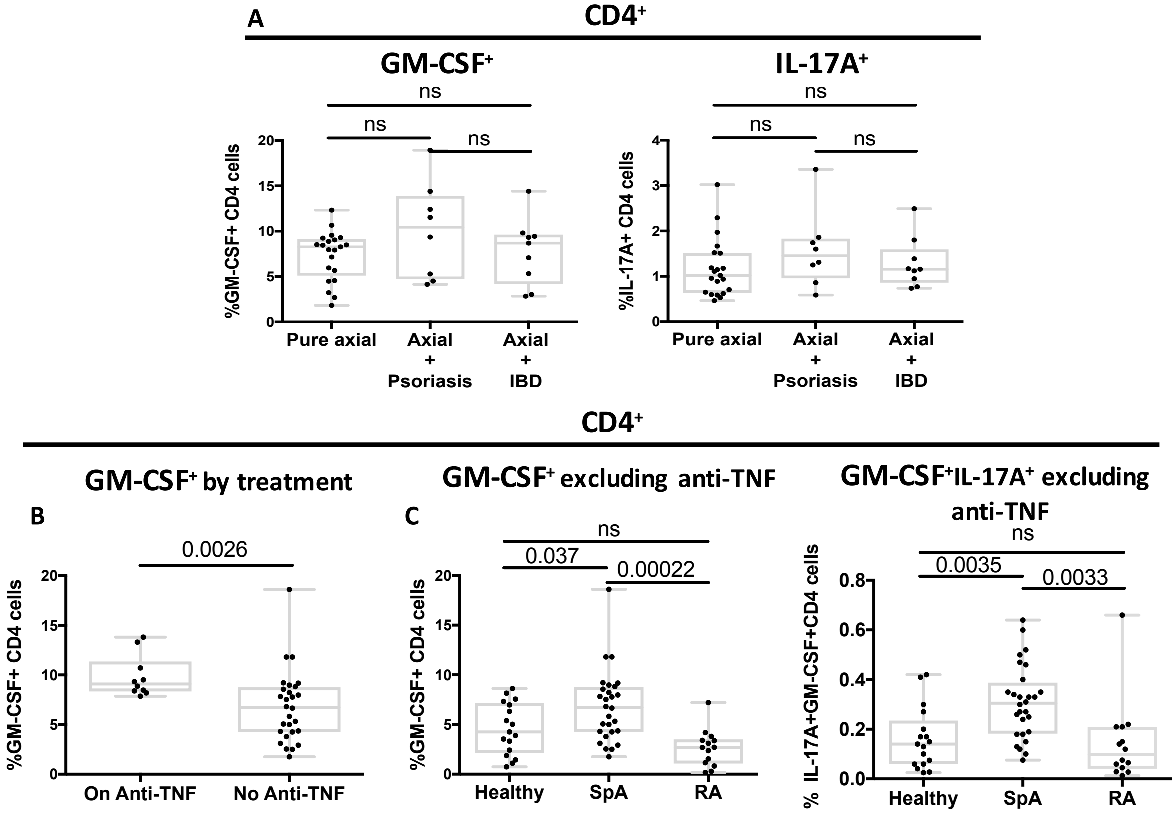

Supplementary Figure 2. CD4 GM-CSF frequency in SpA patients by comorbidity and TNFi treatment. Ex-vivo stimulated PBMCs from patients with axial SpA were gated on $\gamma\delta^-$ $CD3^+CD4^+$. **A** The percentage of $GM\text{-}CSF^+$ and $IL\text{-}17A^+$ cells was analysed according to co-existence of psoriasis (n=8) and inflammatory bowel disease (IBD) (n=9). **B** The percentage of $GM\text{-}CSF^+$ was analysed according to current treatment with TNF-inhibitors (unpaired t test). **C** percentage of $GM\text{-}CSF^+$ (left-hand panel) and $IL\text{-}17A^+GM\text{-}CSF^+$ (right-hand panel) CD4 cells in the TNFi naïve SpA patients (n=28) was compared to RA (n=14) and healthy controls (HC) (n=17). Statistical significance was determined using a Kruskal-Wallis test with p values calculated using Dunn's multiple comparisons test.

Supplementary Figure 3. CyTOF gating, SPADE analysis and validation with flow cytometry

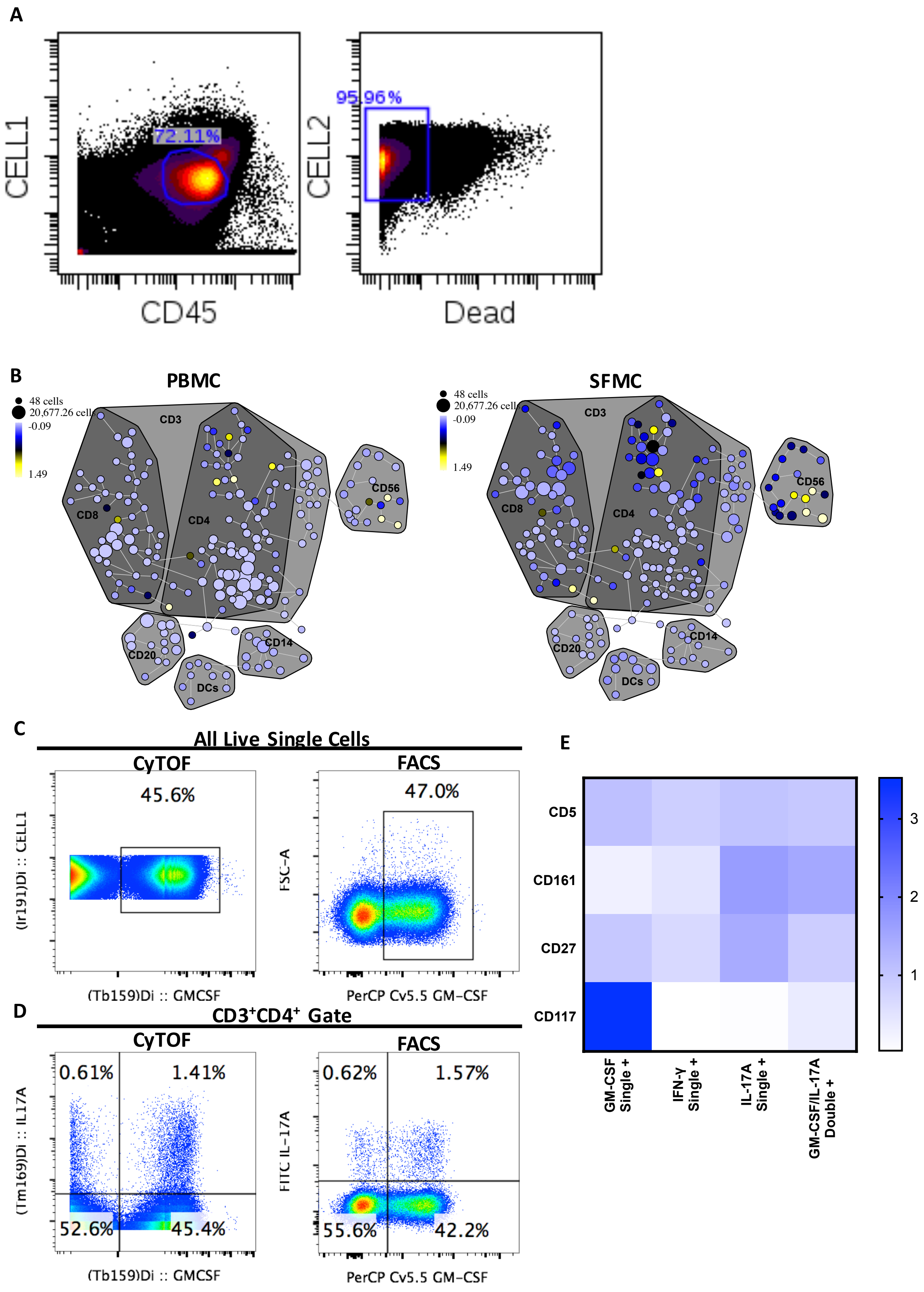

Supplementary Figure 3. **CyTOF gating, SPADE analysis and validation with flow cytometry. A** Representative CyTOF plots generated by cytobank showing gating strategy to identify live single cells from SpA SFMC. **B** Represenatitve SPADE analysis from PBMC and SFMC. Third parameter shown is intracellular GM-CSF. **C** GM-CSF staining of SpA SFMCs gated on all live single cells by CyTOF and FACS from the same patient. **D** GM-CSF and IL-17A staining of SpA SFMCs gated on $CD3^{+}CD4^{+}$ cells by CyTOF and FACS from the same patient. **E** IL-17A, GM-CSF and IFN-γ single-positive and IL-17A/GM-CSF double-positive populations were gated within the $CD3^{+}CD4^{+}$ compartment of SpA PBMCs (n=3) stained by CyTOF and the mean signal of the four surface markers CD5, CD161, CD27 and CD117 was determined in each gated population. Results were normalised to overall expression of each surface marker and presented as a heathmap.

Supplementary Figure 4. Comprison of CD4 GM-CSF and IL-17A/GM-CSF in blood, synovial fluid and tissue.

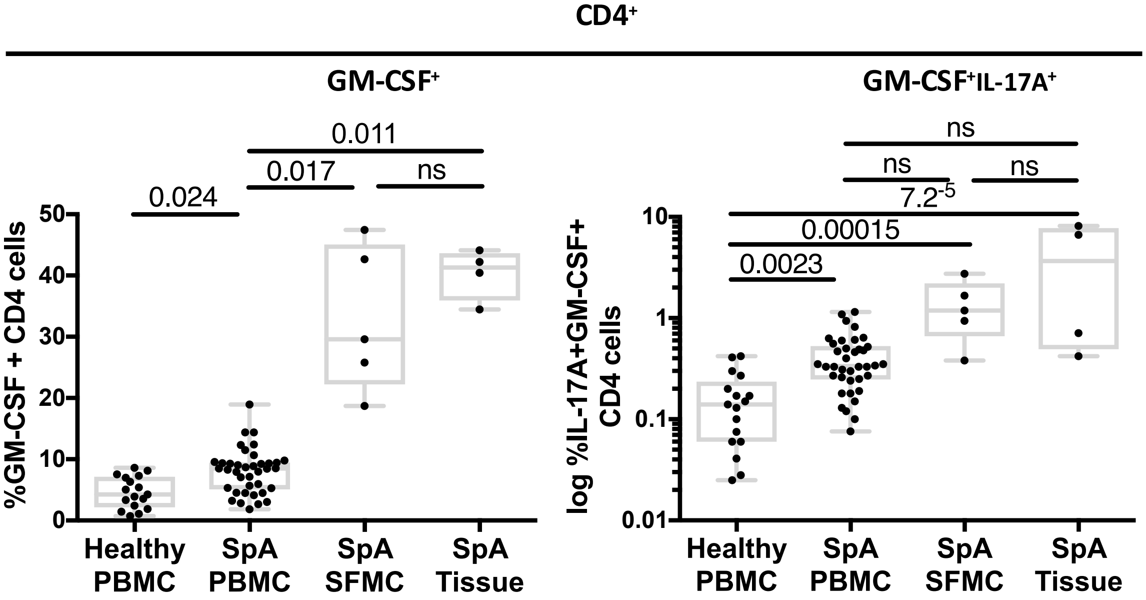

Supplementary Figure 4. Comprison of CD4 GM-CSF and IL-17A/GM-CSF in blood, synovial fluid and tissue. The percentage of GM-CSF^{+} and GM-CSF^{+}IL-17A^{+} cells in ex-vivo healthy PBMC (n=17, as figure 1), SpA PBMC (n=38, as figure 1), SpA SFMC (n=5, as figure 2) and SpA synovial cultures (n=4) was compared. Kruskal-Wallis test with p values calculated using Dunn's multiple comparisons test.

Supplementary Figure 5. Gating strategy for the identification of ILC populations from blood and from tissue explant cultures, and IFN-γ and GM-CSF production by ILCs in healthy and SpA PBMC and inflammatory arthrits tissue explant cultures.

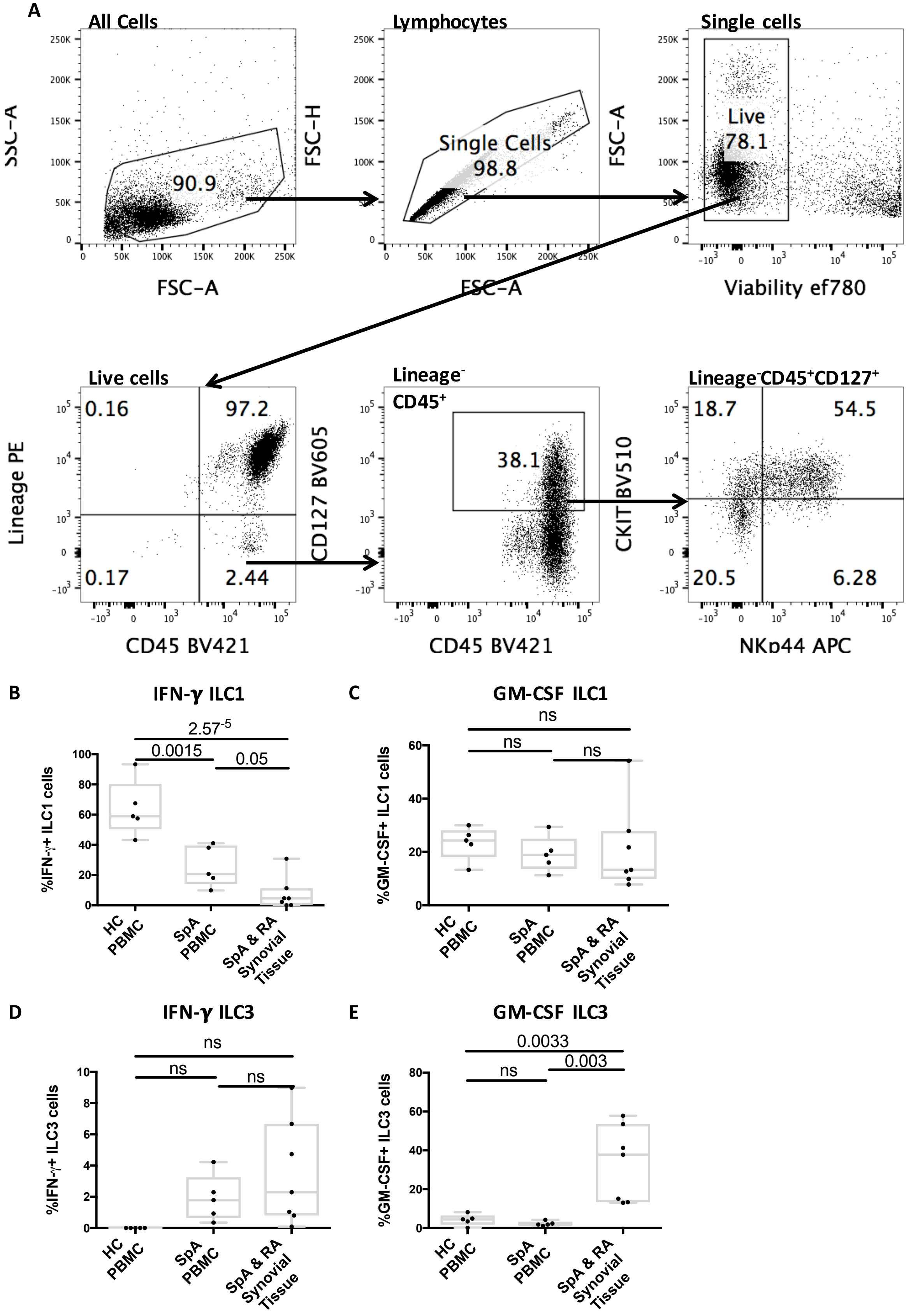

Supplementary Figure 5. Gating strategy for the identification of ILC populations from blood and from tissue explant cultures, and IFN-γ and GM-CSF production by ILCs in healthy and SpA PBMC and inflammatory arthrits tissue explant cultures. Gating strategy for the identification of ILC populations from blood and synovial tissue explant cultures, and IFN-γ and GM-CSF production by ILCs in healthy and SpA PBMC and inflammatory arthrits tissue explant cultures. **A** Gating strategy for identifying innate lymphoid cells from STMC cultures. Lineage negative (Lineage cocktail: CD3, CD5, CD8, CD11b, CD11c, CD19, CD20, CD34, TCR-γδ, CRTH2) CD45 positive cells were then gated on IL7R expression. ILC1 subset defined as $Lin^{-}CD45^{+}IL\text{-}7R^{+}CKIT^{-}$ and ILC3 subset defined as $Lin^{-}CD45^{+}IL\text{-}7R^{+}CKIT^{+}$. ILC3 population further subdivided into NCR^{-} and NCR^{+} subpopulations based on NKp44 expression. **B** and **C** IFN-γ production by ILC1 and ILC3, **D** and **E** GM-CSF production by ILC1 and ILC3, measured by ICS, for healthy controls (n=5), SpA PBMC (n=5) and SpA and RA synovial tissue (n=4+3). ANOVA with multiple comparison correction using a Holm-Sidak test.

Supplementary Figure 6. Triple cytokine capture of CD4 T cells; gating strategy for FACS sorting and RNA seq/qPCR validation of sorted populations

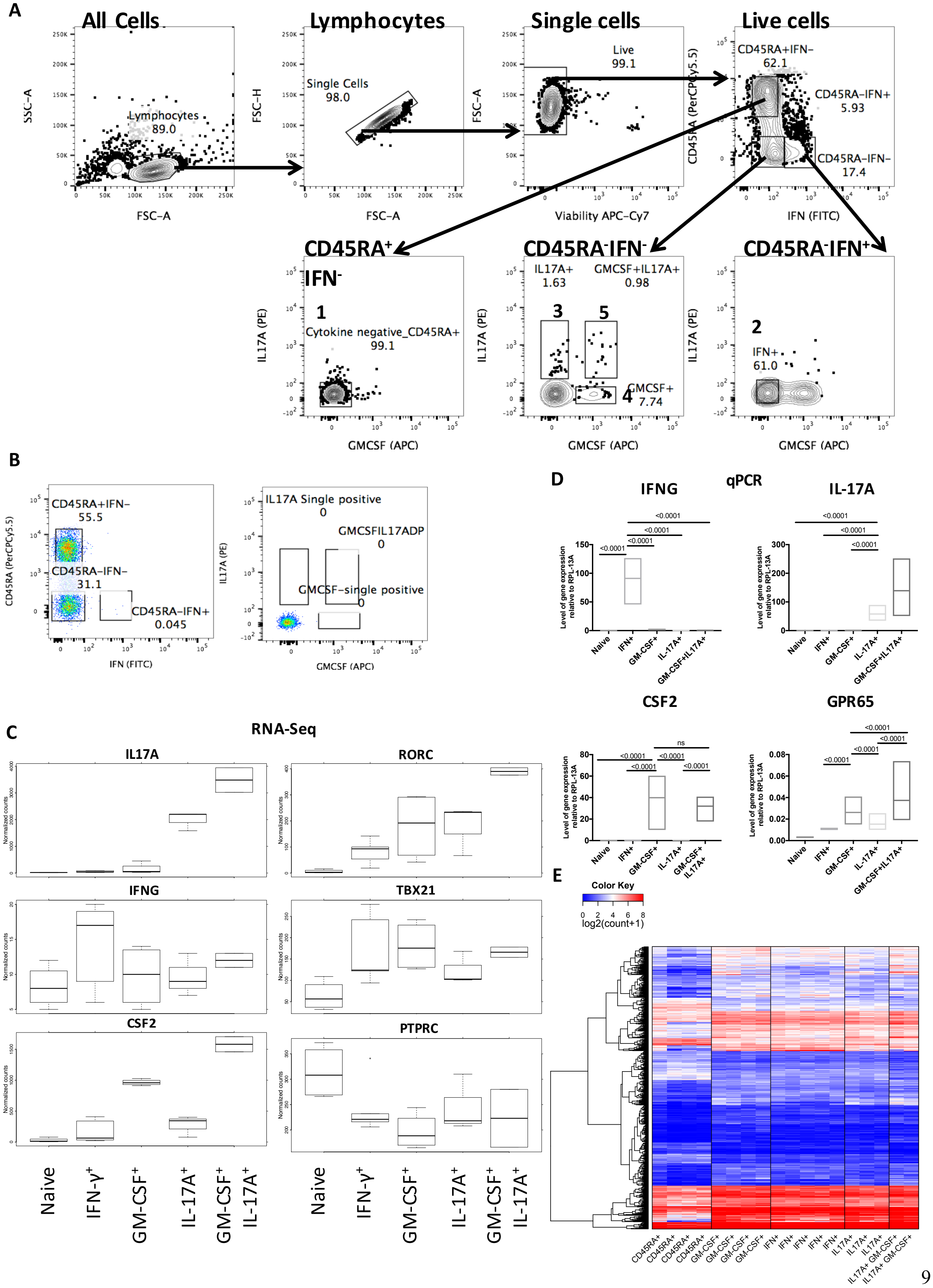

Supplementary Figure 6. Triple cytokine capture of CD4 T cells; gating strategy for FACS sorting and RNA seq/qPCR validation of sorted populations. **A** Gating strategy for triple cytokine capture. PBMCs were obtained from a lymphocyte cone by density centrifugation and CD4 cells were isolated by magnetic bead negative isolation on the same day. The following day cells were stimulated with PMA/Ionomycin and triple cytokine capture was performed for IL-17A, IFN-γ and GM-CSF. 5 populations (numbered 1-5) were sorted according to the gating strategy shown in the representative plots (**A**). Live cells were first gated according to CD45RA and IFN-γ. $CD45RA^{+}IFN\text{-}\gamma^{-}$ cells were then sorted according to a IL-17A/GM-CSF negative gate (population 1: triple –ve CD45RA +ve). $CD45RA^{-}IFN\text{-}\gamma^{+}$ cells were sorted according to a IL-17A/GM-CSF double negative gate (population 2: CD45RA –ve IFN-γ single +ve). $CD45RA^{-}IFN\text{-}\gamma^{-}$ cells were sorted into 3 populations. (population 3: CD45RA –ve IL-17A single +ve; population 4: CD45RA –ve GM-CSF singe positive; Population 5: CD45RA –ve IL-17A/GM-CSF double +ve). **B** Unstimulated control from the same donor. **C** RNA extracted from sorted populations was sequenced and the expression levels of IFNG, IL17A, CSF2 RORC, TXB21 and PTPRC (marker of naïve CD4 cells) genes was plotted for each group of sorted cell. **D** qPCR was used to asses IFNG, IL17A, CSF2 and GPR65 expression of RNA extracted from triple cytokine capture-sorted CD4 cell from a second cohort of healthy donors. Gene expression quantified relative to RPL-13A. Statistical analysis with paired 2-way ANOVA and Tukey correction for multiple testing. **E** Heatmap of all differentially expressed genes with an FDR <0.05 showing all individual samples used.

Supplementary Figure 7. GPR65 silencing and effects of IL-7 on GPR65 expression

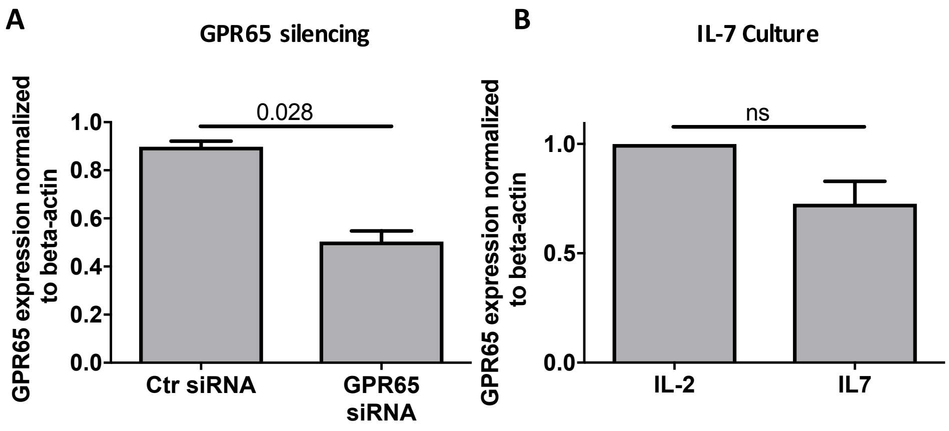

Supplementary Figure 7. GPR65 silencing and effects of IL-7 on GPR65 expression. **A** GPR65 was silenced using a siRNA in primary human CD4 cells and the amount of GPR65 expression was quantified by qPCR post knock-down compared to control siRNA (n=3, mean + SEM, paired t-test). **B** GPR65 expression was determined by qPCR in primary human CD4 cells cultured in the presence of absence of recombinant IL-7 (n=3, mean + SEM, paired t-test).

Supplementary Figure 8. Recombinant IL-7 promotes expansion of GM-CSF but not IL-17A in isolated human CD4 cells and explant tissue cultures. Monocytes are the main population of cells responsive to GM-CSF in SpA blood and joints

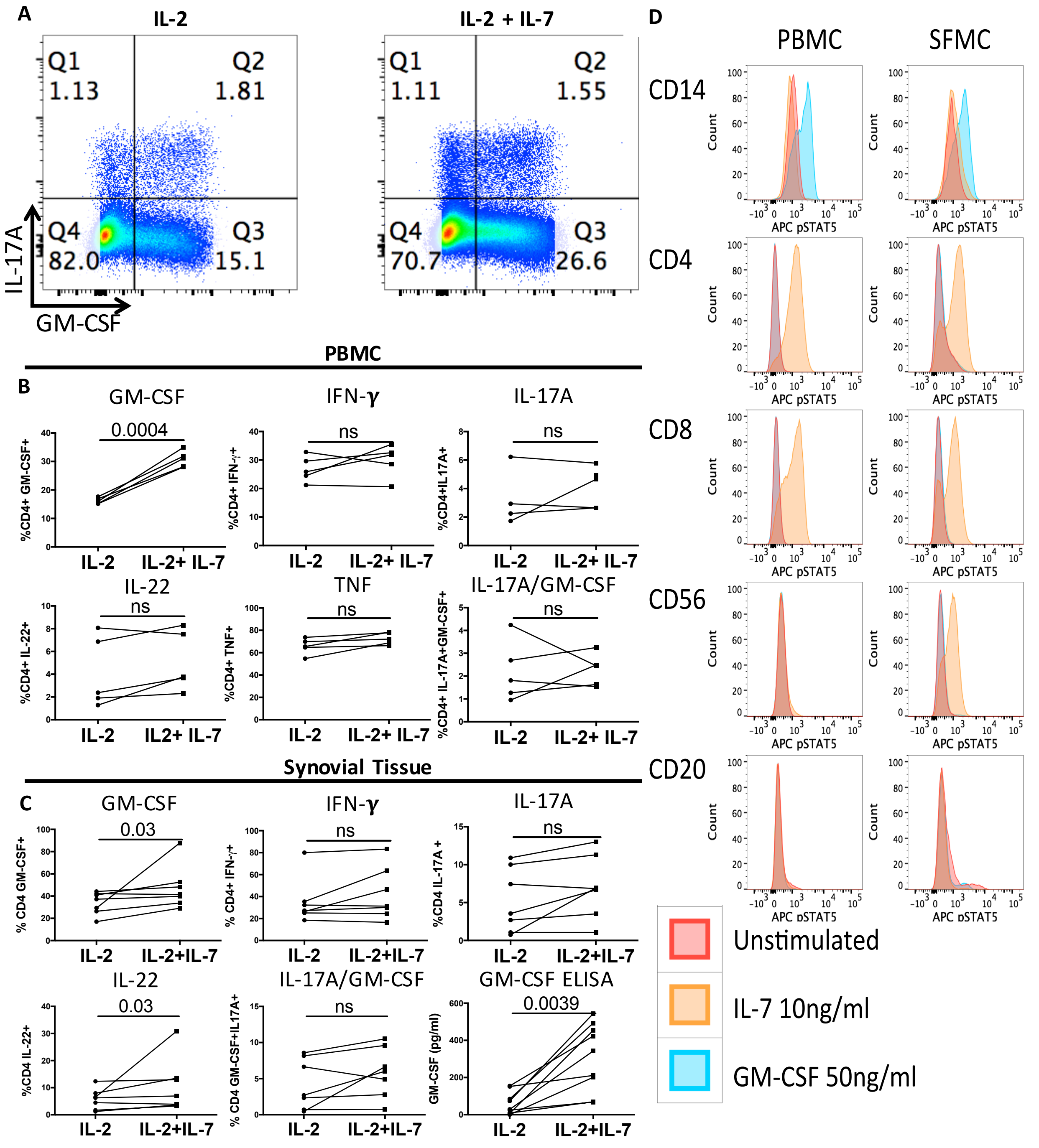

Supplementary Figure 8. Recombinant IL-7 promotes expansion of GM-CSF but not IL-17A in isolated human CD4 cells and explant tissue cultures. Monocytes are the main population of cells responsive to GM-CSF in SpA blood and joints. A Representative flow cytometry plots, gated on live cells, of CD4 cells stimulated for one week in the presence of IL-2 alone or IL-2 with the addition of IL-7. **B** PBMCs from 5 healthy donors were isolated by negative selection and cultured for one week with or without 10ng of recombinant human IL-7. Cells were cultured and activated with anti CD2/3/28 beads and were supplemented with 20 IU/ml of IL-2. At the end of the culture cells were re-stimulated with PMA/ionomycin and intracellular cytokine staining was performed. Statistical significance was determined using a paired t-test. **C** Surgical tissue from 7 patients with inflammatory arthritis undergoing joint replacement surgery was explanted and cultured for two weeks with or without 10 ng/ml of recombinant human IL-7; intracellular cytokine staining of synovial tissue cells was then performed. Data derived after gating on $CD3^+CD4^+$ cells and measurement of GM-CSF by ELISA from culture supernatants (9 independent experiments from 5 patients with inflammatory arthritis). Statistical significance determined using a paired t-test. **D** Matched ex-vivo PBMC and SFMCs cells (n=2) were starved for 4 hours in R0 medium then re-suspend in medium containing recombinant human GM-CSF recombinant human IL-7 for 15 minutes before being placed on ice. Cells were then stained for surface markers followed by an intracellular phospho-STAT5 staining. All events were gated on single then live cells. Live cells were then gated on $CD3^+CD14^-$, $CD14^+CD3^-$, and $CD3^-CD14^-$. $CD3^+CD14^-$ cells were then gated on $CD4^+$ and $CD8^+$ while $CD3^-CD14^-$ cells were gated on $CD56^+$ and $CD20^+$ populations. Phospho-STAT5 signal was plotted in unstimulated (red) and after GM-CSF (blue) or IL-7 (Yellow) exposure as overlaid histograms according to the various cell types.

Supplementary Table 1: Patient and control characteristics peripheral blood cohort

	**Spondyloarthritis (n=38)**	**Rheumatoid arthritis (n=14)**	**Healthy controls (n=17)**
Age, mean (range) years	38 (22-71)	64.6 (49-82)	37.3 (28-62)
Sex, male/female	28/10	6/8	11/6
HLA-B27+ no. (%)	27* (77%)	n/a	n/a
Co-existing known IBD no. (%)	9 (24%)	0	0
Co-existing known psoriasis no. (%)	8 (21%)	0	0
RF/CCP+ no. (%)	n/a	10 (71%)	n/a
BASDAI, mean (range)	3.44 (1-10)	n/a	n/a
DAS28-CRP, mean (range)	n/a	3.38 (1.7-5.33)	n/a
DMARD therapy, current (previous)	6 (0)	13 (0)	n/a
Anti TNF therapy, current (previous)	10 (2)	0	n/a
CRP, mean (range)	17.18 (0.2-98.4)	17.86 (0.7-97.4)	n/a

* unknown HLA-B27 status in 3 AS patients

Supplementary Table 2: Patient and control characteristics for synovial fluid and tissue cohorts

	**SpA matched peripheral blood & synovial fluid (n=5)**	**SpA Synovial Tissue (n=4)**	**RA Synovial Tissue (n=3)**
Age, mean (range) years	38.4 (24-61)	50.5 (23-67)	69 (64-75)
Sex, male/female	2/3	2/2	1/2
HLA-B27+ no. (%)	4 (80%)	2(50%)*	n/a
RF/CCP+ no. (%)	n/a	n/a	3 (100%)
BASDAI, mean (range)	5.84 (4.56-7.1)	3.55(2.62-4.49)	n/a
DAS28-CRP, mean (range)	n/a	n/a	3.4 (3.17-3.7)
DMARD therapy, current (previous)	3(0)	2(2)	3 (0)
Anti TNF therapy, current (previous)	1	2(0)	1(0)
CRP, mean (range)	29.1 (1.8-87)	24.6 (4.9-62.6)	29 (21.2-48.5)

* unknown HLA-B27 status in one SpA patient

Supplementary Table 3. Members of pathways altered in pathway analysis of GM-CSF+CD4 T cells shown Figure 4.

**Pathway**	**Members**
p53 Signaling	*BID, CASP9, THBS1, CD82, PERP, DDB2, SESN3*
VEGF Signaling	*PIK3CD, SPHK2, PTK2, CASP9, MAPKAPK2*
Glycolysis/Gluconeogenesis	*PGAM1, PFKM, PFKP, GALM, GAPDH*
Th17 differentiation	*IL6R, RXRA, TGFB1, LAT, IL23A, IL27RA*
TGF-β Signaling	*TGFB1, THBS1, ACVR1, BMPR1A, SMAD7*
Apoptosis	*PIK3CD, BID, FASLG, CASP9, TNFRSF1A, ERN1*
Sphingolipid Signaling	*PIK3CD, PLCB3, SPHK2, BID, PPP2R3B, TNFRSF1A*
AMPK Signaling	*PFKM, PFKP, PIK3CD, EIF4EBP1, PPP2R3B, AKT1S1*
NK-Kappa B Signaling	*BIRC2, TNFRSF1A, LAT, TRAF3, PLAU*
MAPK Signaling	*FASLG, PPM1B, CACNB1, CACNA2D4, MAPKKAPK2, FLNA, TGFB1, CACNG8, TNFRSF1A*

Supplementary Table 4. Flow cytometry antibodies used

**Antibody Target**	**Fluorochrome**	**Clone**	**Supplier**	**Dilution**
CD3	BV785	UCHT1	BioLegend	1:50
CD4	APC	RPA-T4	BioLegend	1:25
CD4	BV421	RPA-T4	BioLegend	1:50
CD5	PE	UCHT2	BioLegend	1:50
CD8α	BV510	RPA-T8	BioLegend	1:50
CD11b	PE	M1/70.15.11.5	Miltenyi	1:50
CD11c	PE	MJ4-27G12	Miltenyi	1:50
CD14	PE	TÜK4	Miltenyi	1:50
CD19	PE	LNK16	Miltenyi	1:50
CD20	PE	2H7	BioLegend	1:50
CD34	PE	561	BioLegend	1:50
CD45	Pacific Blue	HI30	BioLegend	1:50
CD45RA	PerCP-Cy5.5	HI100	BioLegend	1:50
CD117 (C-Kit)	BV510	104D2	BioLegend	2:50
CD127 (IL-7Rα)	BV605	A019D5	BioLegend	2:50
CD161	BV421	HP-3G10	BioLegend	2:50
CD196 (CCR6)	PE-Cy7	11A9	BD	1:50
CD294 (CRTH2)	PE	BM16	Miltenyi	2:50
TCRδγ	PE	B1	BioLegend	2:50
KIR3DL2	AF647	DX31	In-house	2:50
Viability stain	eFluor780	N/A	eBiosciences	1:250
IL-17A	FITC	eBio64DEC17	eBiosciences	1:50
IFN-γ	AF700	B27	BioLegend	1:100
IL-22	Pe-Cy7	22URTI	eBiosciences	1:50
GM-CSF	PerCP-Cy5.5	BVD2-21C11	BioLegend	1:50
pSTAT5	AF647	47/Stat5(pY694)	BD	1:50
Steptavidin	APC	N/A	BioLegend	1:200
Fixable viability dye	eFluor780	N/A	eBiosciences	1:250

Supplementary Table 5. CyTOF antibodies

**Surface**			
**Tag**	**Target**	**clone**	**concentration (µl/stain)**
170Er	CD3	UCHT1	1
145Nd	CD4	RPA-T4	2
168Er	CD8	SK1	1
144Nd	CD11b	ICRF44	1
148Nd	CD14	RMO52	1
147Sm	CD20	2H7	2
155Gd	CD27	L128	1
154Sm	CD45	HI30	1
153Eu	CD45RA	HI100	1
176Yb	CD56 (NCAM)	NCAM16.2	2
143Nd	CD117	104D2	1
149Sm	CD127	A0195D5	1
164Dy	CD161	HP-3G10	2
141Pr	CD196	G034E3	1
160Nd	TCRgd	B1	2
173Yb	CD11c	BU15	2
151Eu	CD16	3G8	2
171Yb	CD34	581	2
175Lu	CD23	EBVCS-5	2
174Yb	CD336	P44-8	2
**CyTOF intracellular antibodies**			
159Tb	GM-CSF	BVD2-21C11	1
165Ho	IFN-γ	B27	1
142Nd	IL-4	MP4-25D2	1
156Gd	IL-6	MQ2-13AS	1
169Tm	IL-17A	BL168	1
166Er	IL-17F	SHLR17	1
172Yb	IL-21	3A3-N2	1
150Nd	IL-22	22URTI	1
152Sm	TNF-α	MAB11	1
146Nd	IL-2	MQ1-17H12	1
158Gd	IL-10	JES3-9D7	1
